# About the Analysis of 18S rDNA Sequence Data from Trypanosomes in Barcoding and Phylogenetics: Tracing a Continuation Error Occurring in the Literature

**DOI:** 10.3390/biology11111612

**Published:** 2022-11-04

**Authors:** Antonia S. Rackevei, Alyssa Borges, Markus Engstler, Thomas Dandekar, Matthias Wolf

**Affiliations:** 1 Department of Bioinformatics, Biocenter, University of Würzburg, Am Hubland, 97074 Würzburg, Germany; 2 Department of Cell and Developmental Biology, Biocenter, University of Würzburg, Am Hubland, 97074 Würzburg, Germany

**Keywords:** *Trypanosoma*, RNA secondary structure, variable regions, V1–V9, V4, V7/V8

## Abstract

**Simple Summary:**

The variable regions (V1–V9) of the 18S rDNA are routinely used in biodiversity studies. In trypanosome research, more than 70 publications discuss the pitfalls and benefits of the V7/V8 region in trypanosome barcoding and phylogenetics. However, in light of the current 18S rDNA numbering system, V7/V8 of trypanosome research corresponds to V4 in all other organisms (including other Euglenozoa). This misunderstanding is traced back to its origin and corrected for future research.

**Abstract:**

The variable regions (V1–V9) of the 18S rDNA are routinely used in barcoding and phylogenetics. In handling these data for trypanosomes, we have noticed a misunderstanding that has apparently taken a life of its own in the literature over the years. In particular, in recent years, when studying the phylogenetic relationship of trypanosomes, the use of V7/V8 was systematically established. However, considering the current numbering system for all other organisms (including other Euglenozoa), V7/V8 was never used. In Maia da Silva et al. [Parasitology 2004, 129, 549–561], V7/V8 was promoted for the first time for trypanosome phylogenetics, and since then, more than 70 publications have replicated this nomenclature and even discussed the benefits of the use of this region in comparison to V4. However, the primers used to amplify the variable region of trypanosomes have actually amplified V4 (concerning the current 18S rDNA numbering system).

## 1. Introduction

It has long been debated whether ribosomal RNA (rRNA) sequence comparisons are “the Rosetta Stone of phylogenetics” [1] or whether rRNA is the “key to phylogeny” [2]. Over the years, the information obtained either from the primary sequence or the secondary and the tertiary structure was extensively used for phylogenetic studies. Most of these studies focused on the 18S rRNA, especially on its variable regions (V1–V9), which have proven helpful for metabarcoding and phylogenetics in different classes of organisms [3].

The numbering system of the 18S rRNA concerning the primary sequence and the secondary structure is complicated and has changed several times, which has impacted the nomenclature of both conserved and variable regions. Motivated by our interest in using the sequence-structure information of the V7/V8 variable regions to investigate the phylogeny of *Trypanosoma*, we noticed an inconsistency in the nomenclature adopted by the *Trypanosoma* research community and the current numbering system of the 18S rRNA. We emphasize our belief that this does not represent a fault. However, it still is a topic in need of clarification to avoid discrepancies and unfruitful discussions in the literature regarding which variable region could be more critical for metabarcoding. Here, we briefly explain the 18S rRNA nomenclature systems and trace this continuation error in the literature. With this, we expect to contribute to fellow researchers working with 18S rRNA sequences and/or structures.

## 2. Results and Discussion

**Figure 1:**
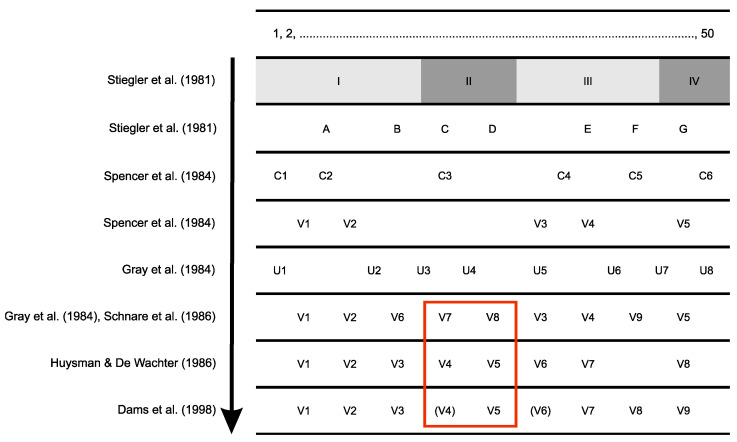
Changes in the small subunit rDNA numbering system throughout the years. The first line shows the helix numbering. Stiegler et al. [4] defined four domains (I–IV) and seven variable regions (A–G). Spencer et al. [5] defined five variable regions (V1–V5) of 18S rDNA that lie between the conserved regions (C1–C6). When V6–V9 were added [6,7,8], V6–V8 came to lie between V2 and V3 and V9 between V4 and V5. V1–V9 regions lie between the universal regions U1–U8 [6]. Huysmans and de Wachter [10] numbered the variable regions V1–V8 consecutively. Dams et al. [11] added the variable region V9. V4 is missing in prokaryotes, and V6 is absent in eukaryotes. Maia Da Silva et al. [27] claimed to use V7/V8, which corresponds to V4 according to the new numbering system.

The first studies on the structure of the rRNA molecules used a simple system of consecutively numbering the helices (e.g., [4]). At the beginning of the 1980s, with the availability of more sequences of small subunit rRNA of different organisms, an effort to identify and classify the structural regions started. At first, four structural domains (I–IV) and seven variable regions (A–G) were defined [4]. Later, a numbering system was adopted for the so-called universal and/or conserved regions (U- and C-regions, respectively) [5,6]. In addition, five variable regions were also identified (V1–V5) [5]. With the discovery and description of four new variable regions (V6–V9) and to avoid changes in the nomenclature proposed in 1984, V6–V9 were placed between the previously described V1–V5. Consequently, V6, V7, and V8 were allocated between V2 and V3, and V9 between V4 and V5 [6,7,8] (Figure 1).

During the development of the European Database on small ribosomal RNA and its variability maps [9], a new numbering system was established, and the nine variable regions (V1–V9) were re-numbered according to the position of the helices (Figure 1). Moreover, this new nomenclature highlighted that one variable region was missing in prokaryotes (V4) and another in eukaryotes (V6) [10,11,12,13,14,15,16,17,18,19].

The system proposed by the European Database on small ribosomal RNA is the most recent and is currently adopted for almost all studies on the structure of 18S rRNA. According to Choi and Park [3], studies on the diversity of eukaryotes noted that the V1–V2, V3, V4, and V9 regions of 18S rDNA had been used to investigate the massive diversity of microbial communities. The V4 (expected amplicon size, 270 bp–387 bp) and V9 (expected amplicon size, 96 bp–134 bp) regions are considered the most popular for metabarcoding. While the V9 region offers the advantage of revealing the extant diversity of eukaryotes (i.e., distantly related species), the V4 is commonly used to evaluate the phylogenetic relationships among them (i.e., closely related species) (cf. [20,21,22,23,24,25,26]).

**Figure 2:**
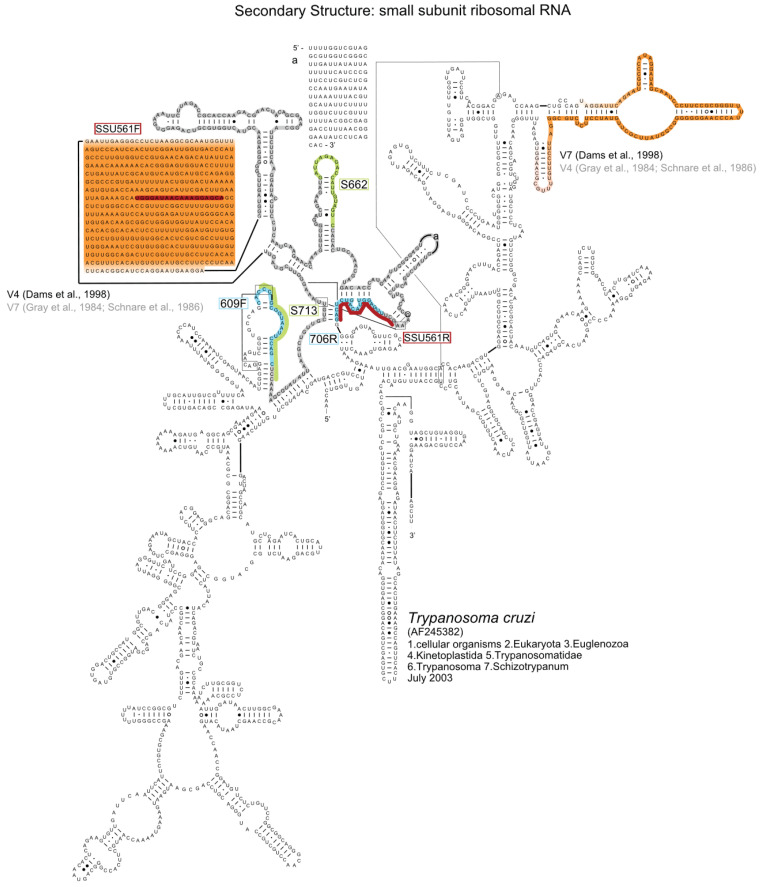
18S rRNA secondary structure of *T. cruzi* obtained from CRW [28]. For regions with pseudoknots, only the primary sequence is shown. The variable regions V4 and V7, according to Dams et al. [11], Gray et al. [6], and Schnare et al. [7], were highlighted in orange and yellow. Primers used in Maia da Silva et al. [27], Noyes et al. [33], and Votýpka et al. [29,30] are highlighted in blue, red, and green, respectively. The sequenced region was highlighted in gray.

Despite that, the majority of the trypanosome research community claims to use V7/V8 regions (Table 1), but a specific numbering system has never been stipulated. Taking into consideration the primers used in different studies, such as 609F and 706R as described by Maia da Silva et al. [27], and the structure of the 18S rRNA of trypanosomes available on the Comparative RNA Website (CRW) [28], we can find the alignment sites and the region of the fragment amplified (Figure 2). According to the current nomenclature (i.e., proposed by the European Database on small ribosomal RNA), the trypanosome V7/V8 region corresponds, in fact, to the V4/V5 region in all other organisms, including other Euglenozoa [17]. Interestingly, three published papers have adopted the updated nomenclature (i.e., V4) for trypanosomes. Two of them have called V4 the region used in the phylogenetic study of avian trypanosomes [29,30] (Figure 2), and another study compared the V4 region of *Trypanosoma brucei* to the V4 region of other eukaryotes [31]. Although using different names to refer to the variable region, all of these studies on trypanosomes are virtually dealing with the same region of the 18S rRNA. Thus, the difference is the adopted nomenclature system but not the variable region itself.

Since the first publication promoting the combination of the variable regions V7 and V8 for trypanosome phylogenetics [27], more than 70 publications have adopted this method and replicated the name of the amplified region as V7/V8. However, as we show in this study, the primers used by the authors have actually amplified V4 (according to the current nomenclature), which is the same region used for all other groups of organisms. Such inconsistency can lead to some confusion, as exemplified by the discussion presented in a review article [32] in which the authors disclaimed that the community of trypanosome researchers uses a different region for barcoding. Nonetheless, it is important to note that despite this nomenclature inconsistency, the validity of the data published was not affected.

By tracing this apparent inconsistency to its origin, we could see that the terminology V7/V8 was systematically established for the phylogeny of trypanosomes, but it does not refer to the current numbering system. To our knowledge, none of the published papers referred to a specific numbering system, which contributes to this continuation error. After clarifying this matter to the scientific community, we suggest that new publications working on fragments of 18S rRNA reference the nomenclature system adopted to avoid future mistakes. By demonstrating that the region used for metabarcoding of trypanosomes is the V4, we hope to close an unbearing discussion on which variable region would be more efficient in investigating the diversity of eukaryotes.

## Figures and Tables

**Table 1:** List of all papers referring to the variable region V4 as the V7/V8 regions of trypanosomes. These papers investigate the diversity of trypanosome species and their phylogenetic relationships.

Authors	Year of Publication	Digital Object Identifier
Maia da Silva et al.	2004	10.1017/S0031182004005931
Rodrigues et al.	2006	10.1017/S0031182005008929
Cortez et al.	2006	10.1017/S0031182006000254
Ferreira et al.	2007	10.1017/S0031182007003058
Maia da Silva et al.	2007	10.1111/j.1365-294X.2007.03371.x
Martins et al.	2008	10.4269/ajtmh.2008.79.427
Viola et al.	2008	10.1017/S0031182008004253
Rodrigues et al.	2008	10.1017/S0031182008004848
Marcili et al.	2009	10.1017/S0031182009005861
Viola et al.	2009	10.1017/S003118200800512X
Marcili et al.	2009	10.1016/j.meegid.2009.07.003
Marcili et al.	2009	10.1016/j.ijpara.2008.09.015
Averis et al.	2009	10.1017/S0031182009990801
Maia da Silva et al.	2009	10.1016/j.actatropica.2008.11.005
Maia da Silva et al.	2010	10.1016/j.meegid.2010.02.005
Cavazzana et al.	2010	10.1016/j.ijpara.2009.08.015
Teixeira et al.	2011	10.1016/j.protis.2011.01.001
Garcia et al.	2011	10.1016/j.ijpara.2011.09.001
Lima et al.	2012	10.1016/j.protis.2011.12.003
Martinković et al.	2012	10.1111/j.1550-7408.2011.00599.x
Hamilton et al.	2012	10.1016/j.ympev.2012.01.007
Ramirez et al.	2012	10.1016/j.exppara.2012.09.017
Borghesan et al.	2013	10.1016/j.protis.2012.06.001
Marcili et al.	2013	10.5402/2013/328794
Lima et al.	2013	10.1186/1756-3305-6-221
Fermino et al.	2013	10.1186/1756-3305-6-313
Silva-Iturriza et al.	2013	10.1016/j.parint.2012.10.003
Marcili et al.	2013	10.1645/12-156.1
Guhl et al.	2013	10.1016/j.meegid.2013.08.028
Acosta et al.	2014	10.1603/ME13177
Marcili et al.	2014	10.1016/j.meegid.2014.04.001
Da Costa et al.	2014	10.4172/ijbbd.1000120
Lemos et al.	2015	10.1186/s13071-015-1193-7
Fermino et al.	2015	10.1016/j.ijppaw.2015.10.005
Juliana et al.	2015	10.1007/s11230-015-9558-z
Lima et al.	2015	10.1186/s13071-015-1255-x
Da Costa et al.	2015	10.1089/vbz.2015.1771
Da Costa et al.	2015	10.1089/vbz.2015.1866
Lima et al.	2015	10.1016/j.actatropica.2015.07.015
Martins et al.	2015	10.1515/ap-2015-0009
Dario et al.	2016	10.1186/s13071-016-1754-4
Attias et al.	2016	10.1111/jeu.12310
Zanetti et al.	2016	10.1016/j.ejop.2016.09.004
Szpeiter et al.	2017	10.1590/s1984-29612017022
Galvis-Ovallos	2017	10.1186/s13071-017-2211-8
Da Costa et al.	2018	10.1590/0037-8682-0098-2018
Ribeiro et al.	2018	10.4269/ajtmh.16-0200
Pacheco et al.	2018	10.1590/s1984-296120180049
Dos Santos et al.	2018	10.1017/S0031182017001834
Espinosa et al.	2018	10.1017/S0031182016002092
Borghesan et al.	2018	10.3389/fmicb.2018.00131
Espinosa-Álvarez et al.	2018	10.1016/j.ijpara.2017.12.008
Suganuma et al.	2019	10.1007/s00436-019-06313-x
Borges et al.	2019	10.1111/jeu.12678
Barros et al.	2019	10.1016/j.ijppaw.2018.12.009
Fermino et al.	2019	10.1186/s13071-019-3463-2
Pérez et al.	2019	10.1186/s13071-019-3726-y
Garcia et al.	2019	10.1007/s10393-019-01440-4
Latif et al.	2019	10.4102/ojvr.v86i1.1634
Kuhls et al.	2019	10.1007/978-1-4939-9210-2_2
Barros et al.	2020	10.3390/pathogens9090736
Garcia et al.	2020	10.1186/s13071-020-04169-0
Rodrigues et al.	2020	10.1016/j.meegid.2019.104143
Boucinha et al.	2020	10.1590/0074-02760200504
e Azevedo et al.	2020	10.1590/0103-8478cr20200262
Marcili et al.	2020	10.1089/vbz.2020.2638
Jaimes-Dueñez et al.	2020	10.1016/j.prevetmed.2020.105159
Dario et al.	2021	10.3390/pathogens10060736
Rosyadi et al.	2021	10.1017/S0031182021001360
Mule et al.	2021	10.1038/s42003-021-01762-6
Dario et al.	2021	10.1016/j.ijppaw.2021.04.003
Ardila et al.	2022	10.1007/s12639-021-01459-x
Yasein et al.	2022	10.29261/pakvetj/2022.034
Chiariello et al.	2022	10.1016/j.ijppaw.2021.11.006
Kostygov et al.	2022	10.1186/s13071-022-05212-y

## Data Availability

Not applicable.
